# Advances in the Role of SIRT3 in Vascular Remodeling in Hypertension

**DOI:** 10.3390/biom16071037

**Published:** 2026-07-16

**Authors:** Abdul Wahid, Md. Tariqul Islam, Md. Sohel Rana, Mst. Morium Parvin, Chunyan Weng, Xiaohong Tang

**Affiliations:** 1 International School of Medicine, Changsha Medical University, 1501 Leifeng Road, Changsha 410219, China; drwahid@csmu.edu.cn; 2 Department of Cardiology of the Third Xiang-Ya Hospital, Central South University, 138 Tongzipo Road, Changsha 410013, China; txh007007@csu.edu.cn; 3 Department of Biochemistry and Biotechnology, School of Biomedical Sciences, Khwaja Yunus Ali University, Sirajganj 6751, Bangladesh; tariqul.bcbt@kyau.edu.bd (M.T.I.); sohel33144@gmail.com (M.S.R.); tasnimm014@gmail.com (M.M.P.)

**Keywords:** hypertension, vascular remodeling, SIRT3, OS, mitochondrial dysfunction

## Abstract

Hypertension-induced vascular remodeling is a major contributor to cardiovascular morbidity and is characterized by endothelial dysfunction, vascular smooth muscle cell phenotypic switching, fibrosis, and inflammation. Sirtuin 3 (SIRT3), a mitochondrial nicotinamide adenine dinucleotide-dependent deacetylase, plays an important role in maintaining mitochondrial homeostasis, regulating redox balance, and modulating cellular energy metabolism. Emerging evidence suggests that SIRT3 deficiency accelerates hypertensive vascular remodeling through multiple mechanisms. In vascular smooth muscle cells (VSMCs), reduced SIRT3 activity enhances mitochondrial reactive oxygen species generation, promotes glycolytic reprogramming, and contributes to phenotypic switching and proliferation. In endothelial cells, SIRT3 mitigates oxidative stress (OS) by regulating the activity of superoxide dismutase 2, thereby preserving nitric oxide (NO) bioavailability and improving vascular function. SIRT3 also suppresses fibroblast-to-myofibroblast transformation by inhibiting the transforming growth factor-β/Smad3 pathway, thereby reducing vascular fibrosis. Furthermore, SIRT3 regulates macrophage metabolic reprogramming and autophagy, inhibits NLRP3 (NOD-, LRR- and pyrin domain-containing protein 3) inflammasome activation, and attenuates vascular inflammation. In perivascular adipose tissue, SIRT3 deficiency exacerbates angiotensin II-induced fibrosis and cytokine secretion, thereby aggravating vascular dysfunction. Collectively, SIRT3 acts as a mitochondrial regulator against hypertension-induced oxidative and inflammatory injury. Targeting SIRT3-dependent pathways may represent a promising therapeutic approach to restore vascular homeostasis and prevent hypertensive vascular remodeling.

## 1. Introduction

Hypertension is a major cardiovascular disorder and an important global public health concern. Its prevalence continues to increase, particularly with population aging [1]. Vascular remodeling is an important pathological process of hypertension, which leads to structural changes such as the thickening of the vascular walls and increased stiffness through pathological changes such as endothelial dysfunction, phenotypic changes in smooth muscle cells, and degradation of the extracellular matrix. SIRT3 is a member of the Sirtuins protein family, a NAD^+^-dependent enzyme that regulates most mitochondrial lysine acetylation and is involved in biological processes such as apoptosis, OS, and energy metabolism [2]. In recent years, studies have found that SIRT3 is involved in hypertensive vascular remodeling by affecting mechanisms such as smooth muscle cell phenotypic switching, endothelial function, and vascular inflammation. This article focuses on the progress of research on SIRT3 in hypertensive vascular remodeling.

Unlike previously published reviews on SIRT3 in cardiovascular diseases, the present review focuses specifically on hypertension-induced vascular remodeling and provides an updated and structured synthesis of recent mechanistic evidence. In particular, this manuscript integrates the latest findings on SIRT3 from multiple vascular cell types, including VSMCs, endothelial cells, fibroblasts, macrophages, and perivascular adipose tissue, to present a unified view of vascular remodeling. Moreover, this review uniquely emphasizes multi-omics evidence (transcriptomics, proteomics, and metabolomics) and systems biology-based pathway integration, which are not comprehensively covered in earlier literature. Therefore, this work provides a more mechanistic and translational perspective on SIRT3 as a central mitochondrial regulator and therapeutic target in hypertensive vascular remodeling.

In contrast to the review published by [3] [DOI: 10.3390/biology12050686], which provides a general overview of SIRT3 in cardiovascular diseases, the present review specifically focuses on hypertension-induced vascular remodeling and further incorporates multi-omics datasets and KEGG/STRING-based network analysis to construct an integrated mechanistic model.

**Figure 1:**
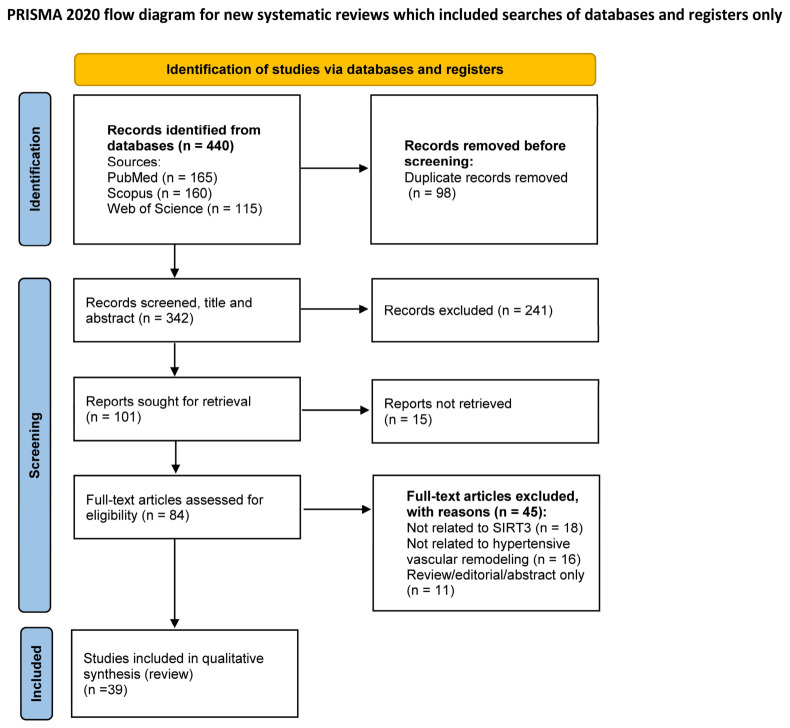
PRISMA 2020 flow diagram illustrating the literature selection process for the review, including identification, screening, eligibility, and inclusion of studies (This work is licensed under CC BY 4.0. To view a copy of this license, visit https://creativecommons.org/licenses/by/4.0/. Accessed on 27 May 2026).

The study selection process followed PRISMA 2020 guidelines to ensure transparency and reproducibility. A total of 440 records were identified from PubMed, Scopus, and Web of Science databases. After removal of duplicates and screening based on title and abstract relevance, full-text articles were assessed for eligibility. Studies meeting inclusion criteria were included in the final qualitative synthesis. The study selection process is presented in a PRISMA flow diagram (Figure 1).

## 2. Overview of Vascular Remodeling

The concept of “vascular remodeling” was first proposed by Baumbach and Heistad in 1989 [4], which is manifested in the course of chronic hypertension when both the inner and outer diameters of the vessel decrease and the ratio of the thickness of the vessel wall to the inner diameter of the vessel increases, while the cross-sectional area of the vessel wall remains unchanged. With the application of pathological morphology, single-cell sequencing, and other technologies, the concept of vascular remodeling has been expanded, and the change in vascular mechanical homeostasis in generalized vascular remodeling can lead to various forms of vascular lumen diameter and vascular cross-sectional area, including increased, shrinkage, or unchanged [5]. Vascular remodeling is an active process that changes the structure and function of blood vessels, involving the proliferation, migration, differentiation, autophagy, apoptosis, remodeling, degradation, and rearrangement of endothelial cells, VSMCs, and fibroblasts. In recent years, several studies have shown that mitochondrial dysfunction is one of the important mechanisms that mediate vascular remodeling, and mitochondria play an important role in vascular endothelial cell injury, smooth muscle cell transdifferentiation, apoptosis, and migration, and may also be involved in regulating the production or degradation of extracellular matrix [6]. Mitochondrial function is mainly dependent on enzymes in its matrix, and the Sirtuin family plays a role in protecting vascular smooth muscle and endothelial cells from harmful effects associated with lipid deposition, OS, and inflammation, and is increasingly being shown to be involved in vascular remodeling [7].

## 3. Overview of SIRTs

The Sirtuins protein family is an NAD^+^-dependent enzyme family whose primary function is deacetylation, which can target histones and non-histones, participate in biological processes such as apoptosis, OS, and energy metabolism, and its enzymatic activity is strictly dependent on nicotinamide adenine dinucleotide (NAD^+^). In 1979, it was first identified in yeast as “silent copulation-type information modulation 2” (SIR2) [8]. Up to now, there are seven members of the SIRTs family: SIRT1 and SIRT2 in the nucleus and cytoplasm, SIRT3, SIRT4, and SIRT5 in the mitochondria, and SIRT6 and SIRT7 are nuclear proteins [9]. The human SIRT3 gene is located at the telomere terminus of chromosome 11 p15.5 (11p15.5), and its gene length is approximately 22 kb (21,902 base pairs), encoding a protein containing 399 amino acids. SIRT3 plays a vital role in maintaining normal mitochondrial biological function through reversible deacetylation of protein lysine. SIRT3 plays a crucial role in cellular homeostasis by regulating multiple mitochondrial substrates involved in energy metabolism, ROS production, and redox balance [10,11]. SIRTs are involved in the pathogenesis of hypertension by regulating vascular endothelial homeostasis, vascular sclerosis, vascular aging, and lipid metabolism [12,13]. In previous studies, SIRT1 and SIRT6 have been studied extensively [14,15]. SIRT3 levels have been found to decline with age, which parallels an increase in the incidence of cardiovascular disease and hypertension [16], and increased expression of SIRT3 attenuates hypertension in the angiotensin II (AngII) hypertension model and the deoxycorticosterone acetate (DOCA)-salt hypertension model [17]. SIRT3 is critically involved in the regulation of hypertensive vascular remodeling [18].

## 4. The Possible Mechanism of SIRT3 Involvement in Vascular Remodeling in Hypertension

### 4.1. SIRT3 and Smooth Muscle Cells

A key feature of vascular remodeling is smooth muscle cell proliferation, migration, and apoptosis. VSMCs typically exhibit a contractile phenotype with little to no proliferation, migration, and extracellular matrix production but switch to a synthetic phenotype after vascular injury (Table 1) [19]. Dedifferentiated VSMCs lose their contractile properties and transform into synthetic, secretory, proliferative, and migratory phenotypes, thereby contributing significantly to the pathogenesis of arterial remodeling [20].

**Figure 2:**
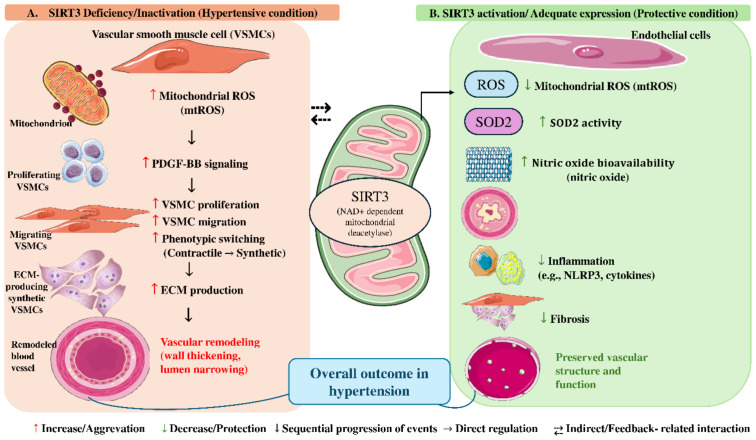
SIRT3 regulates VSMC and endothelial cell function in hypertensive vascular remodeling. (**A**) Under hypertensive conditions, SIRT3 deficiency or inactivation in vascular smooth muscle cells (VSMCs) increases mitochondrial reactive oxygen species (mtROS) production, enhances PDGF-BB signaling, promotes VSMC proliferation, migration, phenotypic switching from a contractile to a synthetic phenotype, extracellular matrix (ECM) production, and ultimately vascular remodeling characterized by wall thickening and lumen narrowing. (**B**) In contrast, adequate SIRT3 expression or activation in endothelial cells reduces mitochondrial ROS, enhances SOD2 activity, improves nitric oxide bioavailability, attenuates endothelial dysfunction, inflammation, and fibrosis, thereby preserving vascular structure and function. The mitochondrion, proliferating VSMCs, migrating VSMCs, ECM-producing synthetic VSMCs, and remodeled blood vessel are labeled for clarity. Red upward arrows indicate increased or aggravated pathological processes; green downward arrows indicate decreased or protective effects; black downward arrows indicate the sequential progression of biological events; solid arrows indicate direct regulatory interactions; black bidirectional dashed arrows indicate reciprocal crosstalk or bidirectional feedback interactions between vascular smooth muscle cells and endothelial cells; and dotted arrows indicate indirect or feedback-related interactions.

The superoxide dismutase (SOD) family consists of three main isoforms: SOD1 (cytosolic Cu/Zn-SOD), SOD2 (mitochondrial Mn-SOD), and SOD3 (extracellular SOD), which play distinct roles in cellular redox regulation. SIRT3 primarily regulates SOD2 through deacetylation, thereby reducing mitochondrial ROS, while also indirectly influencing SOD1 and contributing to extracellular SOD3-mediated vascular antioxidant defense in vascular tissues [21]. Studies have shown that Ang II-induced mitochondrial dysfunction in VSMCs is an important factor in the pathogenesis of hypertension [22]. Mitochondria are the main sites of oxidation and energy conversion in eukaryotic cells, and mitochondrial number and function are important in maintaining the metabolic process of VSMCs. Mitochondrial dysfunction and OS trigger the phenotypic transformation of VSMCs, leading to the occurrence and progression of arterial remodeling [23]. Ang II increases mitochondrial reactive oxygen species (mtROS) production by decreasing SIRT3/SOD2 activity. In contrast, Ecklonia cava extract increases SOD2 activity by upregulating PGC-1a and SIRT3. Then, it reduces mitochondrial DNA damage and mtROS production, thereby improving mitochondrial function in VSMCs and reversing vascular calcification in spontaneously hypertensive rats [24]. ROS can induce the transition of VSMCs to a proliferative phenotype and can also promote the activation of OS-sensitive transcription factors, such as nuclear factor kappa B, and ultimately induce the proliferation of VSMCs [25]. SIRT3 regulates ROS production (Figure 2) and clearance through deacetylation of mitochondrial enzymes, and further mouse models have confirmed that SIRT3 can inhibit angiotensin II-induced vascular smooth muscle cell proliferation in mice (Table 1) [26]. Ang II-induced SIRT3 deficiency leads to enhanced glycolytic flux and increased lactate release from endothelial cells, and elevated lactate levels in the vascular microenvironment stimulate adjacent VSMCs, resulting in a phenotypic shift to a synthetic phenotype [27,28]. SIRT3 deacetylation and activation of long-chain lipoyl-CoA dehydrogenase, a key component of mitochondrial fatty acid β oxidation, fatty acids are an essential component of vascular smooth muscle cell function, SIRT3 inactivation leads to the dysregulation of long-chain fatty acid metabolism, leading to glycolytic transition, down-regulating the expression of the smooth muscle cell marker α-smooth muscle actin, and inducing smooth muscle cell phenotypic switching [16]. In addition, SIRT3 inhibits PDGF-BB-induced vascular smooth muscle cell migration (Figure 2) [25].

Excessive proliferation and phenotypic switching of pulmonary artery smooth muscle cells (PASMC) under hypoxic conditions can lead to the formation of pulmonary hypertension (PAH). Autophagy is the leading cause of pathological vascular remodeling of hypoxic PAH, and SIRT3 overexpression can inhibit the increase in the expression of hypoxia-mediated autophagy-related genes (LC3B-II, BECN-1, and ATG5), while reducing hypoxia-induced PASMC proliferation [29]. Qiu et al. reported that the expression of SIRT3 was significantly down-regulated in the aortic tissue of Ang II-induced thoracic aortic dissection (TAD) mice, which increased vascular rupture and mortality in mice. Deletion of SIRT3 leads to increased ROS accumulation, apoptosis, and secretion of pro-inflammatory cytokines in VSMCs during the development of TAG [30]. In summary, the decrease in SIRT3 expression induced by hypertension leads to the activation of VSMCs, resulting in increased secretion of extracellular matrix and vascular remodeling.

### 4.2. SIRT3 and Endothelial Cells

**Figure 3:**
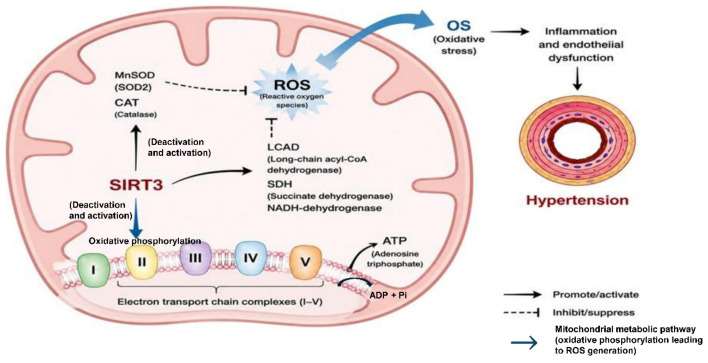
SIRT3-mediated regulation of mitochondrial redox homeostasis in endothelial cells. SIRT3 enhances mitochondrial antioxidant defense by deacetylating and activating MnSOD (SOD2) and catalase (CAT), thereby reducing mitochondrial ROS accumulation. SIRT3 also regulates metabolic enzymes involved in oxidative phosphorylation and fatty acid oxidation, contributing to ATP production while limiting excessive ROS generation. Increased ROS promotes oxidative stress, inflammation, endothelial dysfunction, and hypertension. Solid arrows indicate direct activation; blunt-ended dashed lines indicate inhibition or suppression; and blue arrows represent mitochondrial metabolic pathways associated with oxidative phosphorylation and ROS generation.

Endothelial cell dysfunction is an important cause of hypertensive vascular remodeling [30]. As illustrated in Figure 2B, SIRT3-mediated regulation of endothelial cells complements its effects in VSMCs by reducing mitochondrial ROS, preserving nitric oxide bioavailability, and limiting endothelial dysfunction. SIRT3 is protective in angiotensin II (Ang II)-induced endothelial dysfunction by regulating ROS production [31]. The SIRT3/SOD2 signaling pathway is involved in the occurrence and development of hypertension, and when hypertension is induced by Ang II, compared with normal mice, SIRT3 knockout mice have significantly higher blood pressure, increased SOD2 acetylation, and increased mitochondrial superoxide production, while endothelial nitric oxide decreases. At the same time, the analysis of peripheral blood mononuclear cells of patients with essential hypertension showed a 2.6-fold increase in SOD2 acetylation and a 1.4-fold decrease in SIRT3 levels (Table 1) [32]. SIRT3 inactivation increases mtROS-mediated oxidative stress caused by hyperacetylation of SOD2, leading to endothelial dysfunction and hypertension. In studies on smoking and hypertension, nicotine has also been found to reduce the expression of SIRT3 and promote vascular OS and hypertension [33]. The investigator uses α-linolenic acid (ALA) to restore endothelial autophagic flux and mitochondrial redox stress by increasing SIRT3 activity, thereby improving endothelial cell dysfunction and alleviating experimental hypertension [34]. In addition to regulating ROS levels, SIRT3 is also expressed in endothelial progenitor cells, and in a study, it was found that defective SIRT3/SOD2 signal transduction can cause mitochondrial oxidative damage, resulting in a decrease in the reendothelialization ability of hypertensive endothelial progenitor cells. Knockdown of SIRT3 with siRNA-SIRT3 promoted dysfunction of endothelial progenitor cells, while activation of SIRT3 with ad-SIRT3 enhanced antioxidant capacity and reduced ROS production [35]. Restoration of SIRT3 expression eliminates mitochondrial OS by enhancing SOD2 deacetylation in hypertensive endothelial progenitor cells under hypertensive conditions, and upregulation of SIRT3/SOD2 signaling promotes the reendothelialization of hypertensive endothelial progenitor cells (Figure 3) [36]. As illustrated in Figure 3, SIRT3 maintains mitochondrial redox homeostasis by directly activating the antioxidant enzymes MnSOD (SOD2) and catalase (CAT), thereby enhancing mitochondrial ROS detoxification. In addition, SIRT3 regulates mitochondrial metabolic enzymes involved in oxidative phosphorylation and fatty acid oxidation, which contribute to efficient ATP production while limiting excessive ROS generation. Through these coordinated mechanisms, SIRT3 attenuates oxidative stress, preserves endothelial function, and protects against hypertension-associated vascular remodeling [37]. Therefore, maintaining mitochondrial redox homeostasis in endothelial progenitor cells may be a new target for endothelial injury therapy. As illustrated in Figure 3, mitochondrial oxidative phosphorylation is closely associated with mitochondrial ROS generation, and impairment of this process contributes to oxidative stress and endothelial dysfunction.

### 4.3. SIRT3 and Fibroblasts

**Figure 4:**
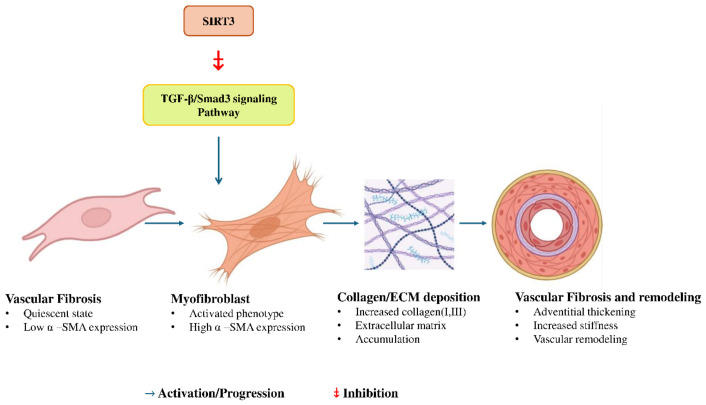
SIRT3 inhibits fibroblast activation and vascular fibrosis via TGF-β/Smad3 signaling. SIRT3 suppresses TGF-β/Smad3 pathway activation, thereby inhibiting vascular fibroblast activation and their differentiation into myofibroblasts. This process reduces extracellular matrix deposition and attenuates vascular fibrosis. The regulation occurs within vascular adventitial fibroblasts and contributes to protection against hypertension-induced vascular remodeling. Red arrows indicate inhibition, whereas blue arrows indicate activation or sequential progression from fibroblast activation to myofibroblast differentiation, collagen/ECM deposition, and vascular fibrosis and remodeling.

Fibroblasts are the main component of the adventitia of blood vessels. When blood vessels are damaged, fibroblasts are activated and transformed into myofibroblasts, which migrate to the middle and intima through the secretion of cytokines, proliferation, and other mechanisms, and participate in and promote vascular remodeling [38]. In a mouse model, knockout of SIRT3 further enhances angiotensin II-induced coronary wall thickening and perivascular fibrosis in hypertensive mice [39]. It may be associated with increased levels of ROS and activation of the transforming growth factor-β/SMAD family member 3 (TGF-β/Smad3) pathway (Figure 4), which has been shown to promote vascular fibrosis and contribute to age-related hypertension [40]. In animal models of pulmonary hypertension, the expression level of SIRT3 in pulmonary adventitial fibroblasts decreased, and SIRT3 overexpression reduced the level of mitochondrial protein acetylation, restored SOD2 activity, reduced glycolysis, improved mitochondrial function, inhibited proliferation and induced apoptosis of PAH pulmonary adventitial fibroblasts [12,13].

### 4.4. SIRT3 and Vascular Inflammation

**Figure 5:**
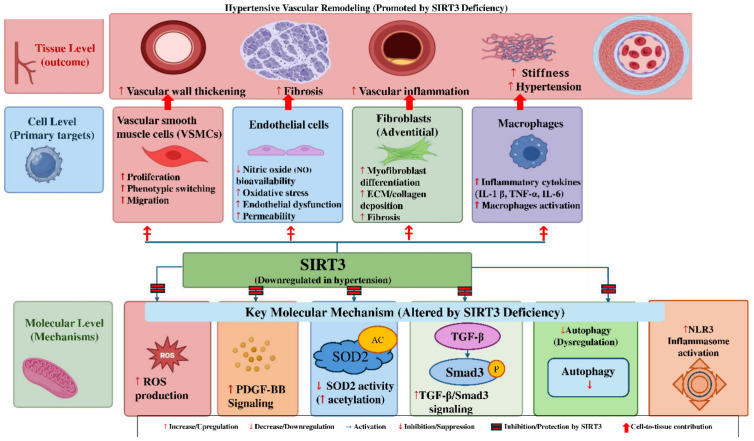
Integrated mechanisms by which SIRT3 deficiency promotes hypertensive vascular remodeling. Reduced SIRT3 activity enhances ROS production, PDGF-BB signaling, TGF-β/Smad3 signaling, and NLRP3 inflammasome activation while decreasing SOD2 activity and autophagy. These molecular alterations promote vascular smooth muscle cell phenotypic switching, endothelial dysfunction with reduced nitric oxide (NO) bioavailability, fibroblast activation, and macrophage-mediated inflammation, ultimately leading to vascular fibrosis, inflammation, wall thickening, increased stiffness, and hypertensive vascular remodeling. Red upward arrows indicate increased activity or upregulation, whereas red downward arrows indicate decreased activity or downregulation. Red inhibitory symbols indicate inhibition or suppression, the red-and-black symbol represent inhibition or protection mediated by SIRT3, and red thick vertical arrows between tissue level and cell level indicate cell-to-tissue contribution. SIRT3 deficiency enhances ROS and PDGF-BB signaling, suppresses SOD2 activity, activates TGF-β/Smad3-mediated fibrosis, and disrupts PPARα/TFEB-dependent autophagy in macrophages, thereby promoting NLRP3 inflammasome activation and vascular remodeling (Figure 5). At the same time, the clinical relevance of SIRT3 depletion was confirmed in arterioles in patients with essential hypertension, showing a 40% reduction in vascular SIRT3 in hypertensive subjects compared to normotensive subjects [17]. Reduced SIRT3 activity leads to SOD2 inactivation and increased mitochondrial ROS, which decreases nitric oxide bioavailability through eNOS uncoupling, resulting in endothelial dysfunction. These findings are summarized in Table 1. These results suggest that SIRT3 depletion triggers macrophage activation and pro-inflammatory cytokine secretion, triggering chronic inflammation and playing a key role in hypertensive vascular remodeling.

Inflammation plays a vital role in the pathological changes in vascular remodeling, and chronic inflammation, mainly caused by monocytes/macrophages, is one of the causes of hypertensive vascular remodeling [41]. Studies have shown that SIRT3 is a key regulator of the macrophage inflammatory response. SIRT3 expression is significantly reduced under Ang II infusion, resulting in macrophage mitochondrial metabolic reprogramming, redox imbalance, and increased secretion of inflammatory cytokines such as TNF-α, interleukins (IL)-1β, and IL-6 [42]. Autophagy plays an essential role in regulating inflammasome formation, initiation, and maintenance of inflammation. SIRT3-deficient macrophages exhibit blockade of PPARα and transcription factor EB (TFEB) signaling pathways, thereby reducing autophagy and activating NLRP3 inflammasomes (Figure 5) [43,44].

SIRT3 deletion promotes vascular inflammation, while increased SIRT3 expression and activity inhibit the expression of inflammatory markers and decrease inflammatory cytokine levels; ROS activates NFkB-mediated vascular inflammation and induces vascular cell adhesion molecule-1 and monocyte chemoattractant protein-1, which in turn leads to vascular dysfunction [45]. In a mouse model, SIRT3 depletion in SIRT3-deficient mice leads to OS caused by SOD2 hyperacetylation, increases HIF1α, decreases endothelial cadherin, stimulates vascular hypertrophy, and increases vascular permeability and vascular inflammation.

**Table 1:** Pathophysiological Roles of SIRT3 in Vascular Remodeling and OS Regulation.

Pathophysiological Aspect	Role of SIRT3	Refs.
SIRT3 and VSMCs	SIRT3 regulates mitochondrial function in VSMCs, reduces ROS production, and inhibits phenotypic switching involved in vascular remodeling.	[19]
Mitochondrial Dysfunction in VSMCs	Ang II–induced mitochondrial dysfunction increases OS, promoting VSMC proliferation, migration, and extracellular matrix remodeling. SIRT3 deficiency exacerbates these effects.	[26]
Endothelial Dysfunction	SIRT3 regulates ROS detoxification via SOD2 deacetylation, maintaining nitric oxide bioavailability and protecting against endothelial dysfunction.	[32]
Vascular Inflammation	SIRT3 suppresses macrophage activation and NLRP3 inflammasome signaling. Its deficiency increases pro-inflammatory cytokines and vascular inflammation.	[43,44]
Fibrosis and ECM Remodeling	SIRT3 reduces fibroblast activation and ROS-mediated fibrosis. Its loss enhances extracellular matrix deposition and vascular stiffening.	[38]
Perivascular Adipose Tissue (PVAT)	SIRT3 deficiency promotes inflammation and fibrosis in PVAT, worsening vascular remodeling through ROS and cytokine signaling.	[46,47]
Autophagy and Cell Survival	SIRT3 maintains autophagy and mitochondrial quality control. Its deficiency impairs mitophagy, increases OS, and accelerates vascular injury.	[39,44,48]

### 4.5. SIRT3 and Perivascular Adipose Tissue

Perivascular adipose tissue (PVAT) refers to the adipose tissue around the blood vessels of the cardiovascular system and the cerebral blood vessels. In addition to adipocytes, there are fibroblasts, macrophages, lymphocytes, etc. Under pathophysiological conditions such as obesity, PVAT will cause dysfunction, promote the infiltration of inflammatory immune cells and local OS, trigger pathological signaling in the blood vessel wall “from the outside to the inside”, lead to the dysfunction of VSMCs and endothelial cells, and promote the development of hypertension [46]. In a mouse model of myeloid SIRT3 knockout, Ang II accelerated inflammation and fibrosis of perivascular adipose tissue, accompanied by NLRP3 inflammasome activation and IL-1β secretion, which not only led to adipose tissue dysfunction and increased type VI collagen expression, but also induced macrophage activation and exacerbated PVAT remodeling [47].

## 5. Integrated Bioinformatic and Multi-Omic Insights into SIRT3 Signaling Pathways

### 5.1. Omics-Based Evidence of SIRT3 Regulation

Recent integrative omics analyses have positioned SIRT3 as a master regulator of vascular oxidative metabolism, linking transcriptomic, proteomic, and metabolomic shifts to hypertensive remodeling. Transcriptomic evidence from the GEO dataset **GSE24752**, which compares peripheral blood gene-expression profiles from patients with essential hypertension and normotensive controls, supports the involvement of oxidative and inflammatory pathways in hypertension. Therefore, this dataset was used as supportive transcriptomic evidence for hypertension-related molecular changes relevant to SIRT3-mediated vascular dysfunction. Because the relevance of GSE136311 to hypertension-induced vascular remodeling was not sufficiently direct, it has been removed from this section [49]. Proteomic profiling confirms reduced SIRT3-dependent deacetylation of SOD2 and LCAD, impairing mitochondrial ROS clearance and fatty acid oxidation [21]. Metabolomic studies reveal elevated succinate and lactate with decreased TCA intermediates, demonstrating metabolic inflexibility typical of mitochondrial dysfunction [49]. Collectively, these omic layers identify SIRT3 as a multifunctional metabolic sentinel that guards against redox imbalance, inflammation, and fibrosis in vascular tissue [49]. Omics-based analyses of mitochondrial biology demonstrate that SIRT3 is a key regulator of metabolic homeostasis [49]. In hypertension, SIRT3 deficiency leads to SOD2 hyperacetylation and increased OS [21].

### 5.2. Pathway and Network Analyses

Network-based analyses using KEGG and STRING databases consistently place SIRT3 at the intersection of redox, metabolic, and autophagic signaling pathways. Four principal axes define its molecular landscape:

**Redox Axis:** SIRT3 → SIRT3 ↑ → SOD2 ↑ → ROS ↓ → AMPK–PGC-1α ↑ [17].

**Inflammatory Axis:** SIRT3 ↓ → NF-κB ↑ → IL-1β ↑ → IL-6 ↑ → NLRP3 ↑ [50].

**Fibrotic Axis:** SIRT3 ↓ → TGF-β/Smad3 ↑ → ECM accumulation ↑.

**Autophagic Axis:** SIRT3 ↑ → AMPK–ULK1–LC3B ↑ → mitochondrial turnover ↑ [51].

Graph-theoretical analysis highlights high-degree centrality of SIRT3, confirming its integrative role as the hub connecting mitochondrial biogenesis with anti-inflammatory and anti-fibrotic defense mechanisms [51]. Network analysis using the STRING database reveals SIRT3 as a central hub protein in mitochondrial signaling networks [50].

### 5.3. Integrative Multi-Omic Model

**Figure 6:**
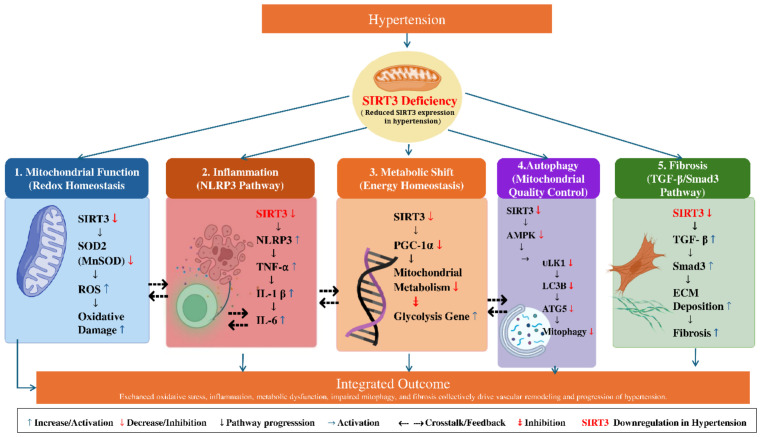
Integrated role of SIRT3 deficiency in hypertensive vascular remodeling. Reduced SIRT3 expression disrupts mitochondrial redox homeostasis, enhances inflammatory signaling, alters metabolic homeostasis, impairs autophagy, and promotes fibrosis. These interconnected mechanisms collectively contribute to vascular remodeling and the progression of hypertension. Solid blue arrows indicate activation or promotion; bidirectional dashed arrows indicate crosstalk or feedback interactions; vertical blue upward arrows indicate increased activity or expression; red downward arrows indicate decreased activity or expression; and blunt-ended lines indicate inhibition.

Integrating transcriptomic, proteomic, and metabolomic data yields a systems-level model describing SIRT3’s regulatory network in hypertensive vascular remodeling. AMPK, PPARα, FOXO3, and SIRT3 interact within reciprocal regulatory networks that coordinate mitochondrial adaptation, antioxidant defense, and autophagy under metabolic and oxidative stress conditions. In this network, SIRT3 can modulate FOXO3-dependent antioxidant gene expression and PPARα/TFEB-dependent autophagy, while AMPK–PGC-1α signaling may also support SIRT3 expression and mitochondrial biogenesis. SIRT3 acts as a mitochondrial deacetylase that directly regulates downstream effectors including MnSOD (SOD2), LCAD, and IDH2, thereby restoring ROS balance and supporting fatty acid β-oxidation (Figure 6). These downstream targets are regulated directly by SIRT3 deacetylase activity and are not upstream regulators of SIRT3. Downstream effectors include SOD2, LCAD, and isocitrate dehydrogenase 2 (IDH2), whose deacetylation restores ROS balance and supports β-oxidation (Figure 6). A positive feedback loop through PGC-1α–SIRT3 signaling enhances mitochondrial biogenesis, while ROS accumulation under SIRT3 deficiency perpetuates NF-κB and TGF-β activation [52]. Machine-learning and Bayesian inference approaches predict that SIRT3 expression serves as a key determinant of vascular resilience, integrating energy metabolism, inflammation, and autophagy into a unified anti-remodeling framework [17]. The proposed multi-omic model thus redefines SIRT3 as a metabolic thermostat—a single molecular node that translates mitochondrial stress into vascular outcomes, offering precision-based targets for antihypertensive therapy [17].

## 6. Future Perspectives and Therapeutic Implications of Targeting SIRT3 in Hypertensive Vascular Remodeling

### 6.1. Therapeutic Activation of SIRT3

Translational strategies increasingly converge on pharmacologic SIRT3 activation to restore mitochondrial redox balance, enhance β-oxidation, and suppress vascular inflammation and fibrosis in hypertension [16].

**Figure 7:**
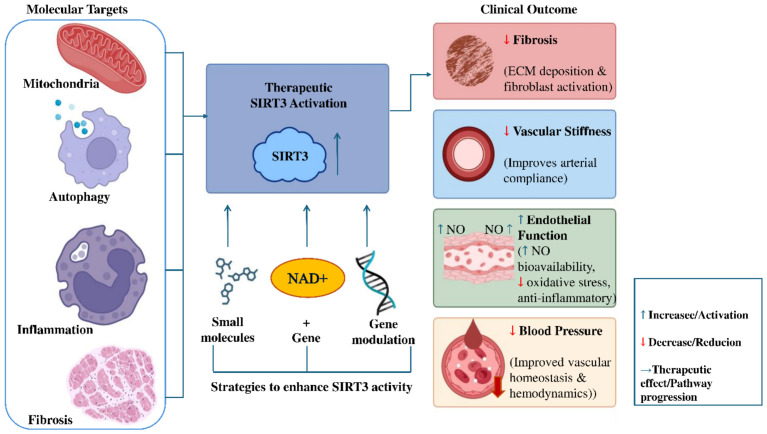
Therapeutic activation of SIRT3 and its effects on vascular remodeling. Therapeutic activation of SIRT3 targets mitochondrial function, autophagy, inflammation, and fibrosis to restore vascular homeostasis. Activation of SIRT3 reduces vascular fibrosis, which subsequently decreases vascular stiffness, improves endothelial function, and ultimately lowers blood pressure. SIRT3 activation also enhances mitochondrial quality control, reduces oxidative stress, and improves vascular metabolic balance, leading to overall attenuation of hypertensive vascular remodeling. Blue arrows indicate activation or therapeutic progression toward SIRT3 activation, whereas downward arrows indicate reductions in pathological outcomes following SIRT3 activation.

NAD^+^ augmentation with precursors such as nicotinamide riboside (NR) or nicotinamide mononucleotide (NMN) replenishes the cofactor pool required for SIRT3 deacetylase activity, deacetylates SOD2 (SOD2) and LCAD, lowers mitochondrial superoxide, and improves endothelial-dependent vasodilation in preclinical models with early human data supporting vascular benefits [16]. Polyphenol-based activators (e.g., resveratrol/pterostilbene) amplify AMPK–PGC-1α–SIRT3 signaling to induce mitochondrial biogenesis, improve nitric-oxide coupling, and blunt NOX-driven OS in vascular cells [53]. Honokiol, a direct SIRT3 agonist, binds the enzyme to boost activity even under partial NAD^+^ depletion, recovering endothelial nitric oxide bioavailability, lowering systolic pressure, and improving arterial compliance in hypertensive rodent studies, thereby offering a mechanism-targeted option that bypasses exclusive dependence on NAD^+^ boosting [16,53]. Beyond classical small molecules, nutrient-based interventions (Mediterranean/ketogenic patterns, intermittent fasting) elevate cellular NAD^+^, engage NAMPT–AMPK signaling, and reinforce SIRT3-mediated metabolic reprogramming, suggesting diet–drug combinations as pragmatic precision therapies for vascular remodeling [54]. Collectively, these approaches position SIRT3 activation as a multi-modal therapeutic axis—spanning pharmaceuticals, nutraceuticals, and lifestyle—capable of repairing mitochondrial function while simultaneously attenuating inflammatory and fibrotic cascades that sustain hypertensive vascular injury (Figure 7) [16,53,54,55].

### 6.2. Gene and Epigenetic Modulation

Gene-level restoration of SIRT3 using CRISPR/Cas-based activation or viral overexpression normalizes mitochondrial membrane potential, suppresses ROS, and reverses pro-fibrotic and pro-proliferative transcriptional programs in endothelial and smooth muscle cells, indicating the feasibility of targeted reconstitution in diseased vasculature [53]. At the epigenetic layer, chronic vascular stress and aging drive SIRT3 promoter hypermethylation and histone H3 lysine 9 (H3K9) hypoacetylation, recruiting DNMT1/HDAC3 to silence transcription and locking cells into a high-ROS, low-ATP state that accelerates remodeling [16,56,57]. Reversal strategies include CRISPR-dCas9–TET1 demethylation of the SIRT3 locus, HDAC inhibition (e.g., trichostatin A, valproate, entinostat) to relax chromatin, and siRNA against DNA methyltransferase 1 (DNMT1)/histone deacetylase 3 (HDAC3)—each reopening antioxidant and mitophagy programs while restoring SIRT3-dependent deacetylation of SOD2/IDH2/LCAD [55,56,58]. Non-coding RNAs integrate metabolic and inflammatory signals with SIRT3 repression: miR-34a/miR-195/miR-214 downregulate SIRT3 translation during OS, whereas lncRNA-mediated scaffolding can stabilize SIRT3 expression; antagonizing these miRNAs or enhancing protective lncRNAs reinstates mitochondrial quality control and endothelial resilience [57,58,59]. The aging–NAD^+^–SIRT3 axis provides a unifying framework: NAD^+^ decline with age diminishes SIRT1/FOXO3a/PGC-1α inputs to the SIRT3 promoter, compounding epigenetic silencing and promoting vascular stiffness—an interplay that is pharmacologically reversible via NAD^+^ repletion plus epigenetic editing [16,55,57].

### 6.3. Predictive Biomarkers and Clinical Translation

A SIRT3-anchored biomarker panel can operationalize precision therapy by linking mitochondrial deacetylation status to vascular function in real time, enabling patient selection and response monitoring [16,53]. Circulating SOD2 activity and the acetyl-SOD2 ratio reflect SIRT3 deacetylase activity and mitochondrial ROS detoxification capacity, providing mechanism-proximal readouts for trials of NAD^+^ boosters or honokiol [16,55]. Plasma NAD^+^/NADH ratio, protein acetylation index (Ac-Lys), and plasma lactate quantify systemic metabolic shifts toward oxidative phosphorylation and away from glycolysis following SIRT3 activation, aligning biochemical correction with clinical improvement in stiffness and blood pressure [16,54,55]. Epigenetic signatures—SIRT3 promoter methylation, global LINE-1 methylation, and H3K9ac/H4K16ac—serve as tractable markers of chromatin accessibility at the SIRT3 locus, complementing metabolic biomarkers to capture nuclear-mitochondrial crosstalk during therapy [55,56,57]. Circulating microRNAs (miR-34a, miR-195, miR-214) and exosomal miRNAs derived from skeletal muscle or adipose sources offer minimally invasive indicators of systemic SIRT3 repression and inter-organ signaling that exacerbates vascular remodeling, expanding diagnostic reach beyond the vessel wall [59,60]. For clinical endpoints, integrating biomarker shifts with flow-mediated dilation, pulse-wave velocity, carotid intima-media thickness, and blood pressure trajectories will validate SIRT3-centric interventions and support regulatory pathways for combination regimens (NAD^+^ precursors ± HDAC inhibitors ± direct SIRT3 agonists) (Figure 7) [16,53,55].

Ultimately, a trial-ready framework emerges: baseline SIRT3 methylation/miRNA/NAD^+^ profiling to stratify “low-SIRT3” hypertensive phenotypes; induction with NR/NMN or honokiol to restore deacetylation; adjunct epigenetic editing or HDAC inhibition for persistent silencing; and longitudinal multi-omic monitoring to titrate therapy for maximal vascular remodeling reversal with minimized off-target effects [16,53,55,56,57].

## 7. Conclusions

In summary, SIRT3, a key regulator of mitochondrial function, is primarily involved in hypertensive vascular remodeling through its effects on various mechanisms, including smooth muscle cell phenotypic switching, endothelial function, and vascular inflammation. Further preclinical and clinical studies are needed to confirm the therapeutic potential of SIRT3 targeting.

## Data Availability

No new data were created or analyzed in this study. Data sharing is not applicable to this article. All information discussed in this manuscript is available from the published sources cited in the reference list.
